# A day-by-day investigation of changes in criminal convictions before and after entering and leaving opioid maintenance treatment: a national cohort study

**DOI:** 10.1186/1471-244X-13-262

**Published:** 2013-10-16

**Authors:** Anne Bukten, Jo Røislien, Svetlana Skurtveit, Helge Waal, Michael Gossop, Thomas Clausen

**Affiliations:** 1 SERAF–Norwegian Centre for Addiction Research, University of Oslo, Kirkveien 166, N-0407, Oslo, Norway; 2 Department of Biostatistics, Institute of Basic Medical Sciences, University of Oslo, Oslo, Norway; 3 Department of Pharmacoepidemiology, Division of Epidemiology, Norwegian Institute of Public Health, Oslo, Norway; 4 Kings College London, National Addiction Centre, London, UK

## Abstract

**Background:**

Opioid maintenance treatment (OMT) is associated with reduced crime among heroin users, but little is known about how crime changes during different phases of treatment. The aim of this study was to investigate changes in criminal convictions on a day-to-day basis before and after entry or discharge from OMT.

**Methods:**

National cohort study of all patients (n = 3221) in OMT in Norway 1997-2003. Patients were followed over a 9-year period, before, during, and after treatment. Criminal convictions were studied on a day-to-day basis in relation to treatment status. A time-continuous estimate of the probability of convictions within the population for all days during observation was calculated.

**Results:**

Changes in convictions were evident *before* changes of treatment status. During the 3 years prior to OMT, the convictions rate was approximately 0.4% per day. Prior to OMT, convictions decreased to about 0.2% per day on the day of treatment initiation. During the weeks before dropping out of treatment, convictions increased. The patterns during periods of transition were the same across gender, age and pre-treatment conviction-levels.

**Conclusions:**

Changes in convictions often occurred prior to changes in treatment status. Reductions in criminal convictions were found in the period before entry (or re-entry) to OMT, and increases in criminal activity were found in the months prior to treatment interruption.

## Background

In treating heroin-dependent persons, opioid maintenance treatment (OMT) combined with psychosocial support has been found to be effective [1,2], and has been linked to improvements in a number of outcomes, including heroin use [3,4], mortality [5-8] and criminal activity [9-15]. OMT is often considered a long-term treatment. However, attrition and relapses are major challenges in OMT; treatment compliance is often poor and dropout from treatment is common [16,17]. Patients who cycle in and out of treatment typically show less improvement during treatment [9,18] and relapse to more drug use and criminal activity when out of treatment [4,9,19]. For patients with several treatment episodes, the high-risk periods outside of treatment are of special concern.

Studies examining the effects of OMT on criminal activity have typically compared levels of criminal involvement which have been aggregated for specific blocks of time, e.g., the period before treatment, during treatment and after treatment [9,20], and the mean total amount of criminal involvement in one period is compared with that in another period. However, categorizing the time axis into blocks of time effectively removes an important variable describing outcomes in the dynamic process of treatment, namely the continuity of change over time. Hence, comparing total amounts of crime aggregated for time periods does not optimally describe the time-varying and continuous probability of criminal activity *within* the different phases inside and outside of treatment. Because drug users move through different stages of both addiction and treatment, a more sensitive longitudinal approach may be required to identify and understand key factors influencing drug use and treatment over time [20,21].

Although a more time-continuous approach has been used for investigating high-risk periods of mortality associated with the changes in treatment status, such studies have typically considered the weeks immediately after treatment induction and cessation, and compared these short-time risk intervals with the average risk over longer time periods [6,21]. In contrast, our study compares the criminal activity during all days before, during and after treatment as a time-continuous variable.

The present study investigates criminal convictions on a day-to-day basis and over prolonged periods of time for all days before, during and after treatment. The main purpose of the study was to investigate on a day-by-day basis the criminal convictions in a national Norwegian cohort of patients in opioid maintenance treatment. More specifically, the study investigates changes in criminal convictions during the time periods before and after entering, re-entering, and leaving treatment.

## Methods

Nationwide registers on all patients (n = 3221) who entered OMT in Norway were cross linked with official data from the Norwegian crime statistics (Statistics Norway) using the unique 11-digit identification number, assigned by the Norwegian state for all citizens. The Norwegian crime statistics comprised detailed information of all cases registered by the police, including the date of the criminal incident and offence details. The OMT patient records included information on age and gender, and the exact date of treatment entries, re-entries, and discharges.

### Study setting

In Norway, OMT is only available through a single publicly funded programme that is provided in all treatment centres and which has a monopoly in respect to OMT admissions [22]. The programme has been available on a national level since 1998, and is integrated into the general health and social service systems [23]. In the study period, patients applied to OMT on a standardized form via their social service centre, and applications were registered in the regional OMT centre [22]. The Norwegian model can be characterized as rehabilitation oriented [22], consisting of both non-medical interventions targeted at housing and psychosocial aspects as well as the maintenance medication. The general practitioner (GP) plays an important and integrated role in the Norwegian OMT system. In 2010, 68% of patients were prescribed OMT medications by their GP although all treatment initiations were conducted at the regional OMT centres [24]. At the time of our observation period, the OMT programme could be considered as relatively high threshold and restrictive: the criteria for admission specified that patients should be 25 years of age, dependent on heroin for several years, and to have experienced prior abstinence-oriented treatment, though interpretation of these criteria was not rigid [22].

Termination of treatment could be either voluntary or involuntary. The absolute criteria for involuntary terminations were violence and threats, but treatment could also be terminated in cases of continued substance abuse, lack of treatment cooperation and lack of rehabilitation efficacy [25]. Very few discharges were made on the basis of the absolute criteria. A few patients decided to leave treatment voluntarily because they considered treatment a success and wanted to become drug-free, while most patients simply dropped out of treatment by discontinuing their attendance of the programme. Dropout for reasons related to convictions, for example incarceration, are considered to be unlikely. According to the national guidelines at the time of observation, patients in OMT could receive OMT-drugs in prison, during periods on remand or short sentences. Following potential treatment termination, the only possibility to obtain access to OMT was to apply for readmission [22].

### Participants

All patients (n = 3221) in Norway who started OMT from September 15^th^ 1997 until December 31^st^ 2003 were included. The study period consisted of varying observation periods for each individual; from admission to OMT until the last day of observation (December 31^st^), creating a dynamic cohort. The pre-treatment period was defined as the three years prior to each individual’s treatment start.

Some patients (n = 135) died between treatment start and the last day of observation, and their observation time was censored at the date of death. “Treatment start” is the date first receiving maintenance medication with either methadone or buprenorphine.

The Norwegian crime statistics provided data on date of the criminal event, penal code and four prosecuting decisions: formal charge leading to conviction, formal charge leading to acquittal, fines, and other. All convictions are decisions finding a person guilty of a crime in the court of law. In our study, only formal charges leading to convictions were included in the analysis. A description of types of convictions that the cohort accounted for can be found in previous publications [14,20,26].

A person may have had several convictions during a single day, e.g. being convicted for both stealing a car and for driving under the influence. In our study, the unit of observation was a ‘crime-day’, defined as a day an individual had one or more criminal offences leading to convictions. The crime-day represents the day that the crime was committed.

### Patient subgroups

In addition to an overall estimate of criminal convictions in the cohort, data were stratified according to gender, age and pre-treatment criminal activity. For age, patients were categorized into three groups according to age at treatment start; below 30 (n = 450), 30-40 (n = 1797), and above 40 years (n = 974). Patients were similarly divided into three groups according to levels of convictions during the 3 years prior to treatment start; patients with no convictions (n = 1375), 1-27 convictions (n = 1659), and more than 27 convictions (n = 187), comprising the 90-percentile of patients having the most criminal convictions during the pre-treatment period. These categories were labelled the “no conviction group”, “medium conviction group” and “high conviction group”, respectively.

All patients were included when estimating the probability of criminal convictions at the time of entry to the first treatment episode. When calculating rates of criminal convictions during the period of treatment drop-out, all patients who dropped out of treatment at least once (n = 1175) were included in the analysis. All patients having several individual treatment episodes (n = 515) were included when calculating rates of criminal convictions during the period prior to treatment re-entry.

### Ethics

The study was approved by the Regional Committees for Medical and Health Research Ethics, The Norwegian Social Science Data Services (NSD), the Norwegian Directorate of Health and the Police Directorate. Files were merged and made anonymous by Statistics Norway.

### Statistical analysis

Data are presented as mean (SD) or n (%). Continuous variables were compared using t-tests and discrete variables using chi-square tests. The number of crime-days at a specific given time, measured relative to entering, re-entering or leaving treatment, divided by the number of patients observed at that time, is an estimate of the probability of a criminal activity at that time [27]. Calculating this probability for all times, i.e. all days when patients were observed, provides a time continuous estimate of the probability of criminal in the cohort on each day. Two essential times; “date of treatment entry (or re-entry)” and “date of treatment termination” were used as reference dates for which the day-by day probability of criminal convictions was calculated both before and after. In order to reduce the effect of natural day-to-day variation, the estimate was smoothed using a Gaussian Kernel [28]. A time continuous 95% confidence interval (CI) was calculated using bootstrapping [29], a general statistical re-sampling methodology. Statistical analyses were performed in R version 2.12 and Stata version 11.

## Results

The patients in the cohort had a mean age of 37 years at treatment entry and the majority (67.6%) were men (see Table 1). Women were significantly younger than men (35.5 vs. 37.7 years) at entry to OMT, and spent slightly longer time in treatment than men. No differences were found between men and women in likelihood of dropping out of treatment or in having several treatment episodes.

**Table 1:** Demographics for OMT patients in Norway (1997-2003) (n = 3221)

**Characteristics of patients and treatment episodes**	**All patients**	**Male**	**Female**	**P-value**^**a**^
**Patients (n, %)**	3221 (100)	2176 (67.6)	1045 (32.4)	
**Age at treatment start (mean, SD)**	37.0 (6.7)	37.7 (6.6)	35.5 (6.6)	< 0.001
**Age groups (n, %)**				
*< 30*	450 (14.0)	254 (11.7)	196 (18.8)	< 0.001
*30-40*	1797 (55.8)	1185 (54.5)	612 (58.6)	< 0.001
*> 40*	974 (30.2)	737 (33.9)	237 (22.7)	< 0.001
**Continuous treatment (n, %)**	2046 (63.5)	1391 (63.9)	655 (62.7)	0.492
**Dropout (n, %)**	1175 (36.5)	785 (36.1)	390 (37.3)	0.492
*1 treatment episode*	660 (20.5)	440 (20.2)	220 (21.1)	0.584
*2 treatment episodes*	434 (13.5)	294 (13.5)	140 (13.4)	0.929
*3+ treatment episodes*	81 (2.5)	51 (2.3)	30 (2.3)	0.371
**Months in waiting-list, (median, SD)**	5.30 (11.78)	5.49 (11.84)	4.90 (11.65)	0.099
**Years in treatment, continuous (median, SD)**	1.97 (1.52)	1.94 (1.52)	2.05 (1.55)	0.059
**Years in treatment, drop out (median, SD)**	1.26 (1.28)	1.22 (1.21)	1.36 (1.39)	< 0.05
**Months between treatment (median, range)**^**b**^	2.7 (0-51.9)	3.2 (0-51.9)	2.4 (0-50)	0.187
**Months after treatment (median, range)**^**c**^	13.1 (0-58.9)	13.1 (0.2-58.9)	13.2 (0-50.5)	0.791

^a^P-value for gender difference. ^b^Between treatment period: sum of months between treatment episodes. ^c^The post-treatment period: sum of months after ending treatment.

**Figure 1:**
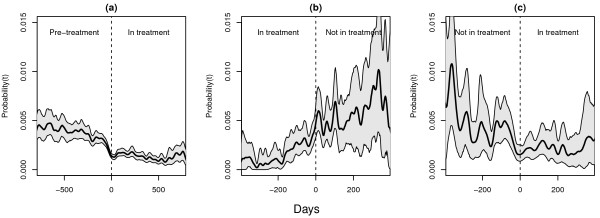
**Day-to-day rates of criminal convictions.** Day-to-day rates of criminal convictions (95% CI) for all patients **(a)** entering OMT for the first time, **(b)** leaving OMT and **(c)** re-entering OMT. **a:** X-axis; days before and days after first treatment entry (n=3221). Y-axis; the day-to-day rate of criminal convictions. **b:** X-axis; days before and days after treatment drop out (n=1175). Y-axis; the day-to-day rate of criminal convictions. **c:** X-axis; days before and days after treatment re-entry (n=515). Y-axis; the day-to-day rate of criminal convictions.

Prior to first treatment entry, the rate of criminal convictions was generally high, with a daily rate of criminality within the cohort of approximately 0.4% per day (Figure 1a). However, about three months prior to treatment initiation, the rate of criminal convictions gradually fell to a lower level, at about 0.2% per day at the day of treatment initiation. This lower level was maintained throughout most of the treatment period (Figure 1a).

During the two months prior to treatment termination the rate of convictions again gradually rose, reaching a higher level around the day of treatment dropout. This increased level prior to treatment dropout is somewhat higher than the pre-treatment level but with wide confidence intervals due to fewer observations with 35% (n = 1175) of patients dropping out at least once (Figure 1b). For those drug users who re-entered treatment, this higher crime level after leaving treatment was continued until approximately three months before re-entering treatment, when it again fell to a lower level of about 0.2% (Figure 1c). Compared to the period prior to the first episode of treatment, the rate of criminal convictions was higher during the period before re-entering OMT.

There was a slight gender difference with a significantly higher level of criminal activity among men as compared to women. Consequently the differences between in-treatment and out of treatment periods were less marked among women. When data were stratified by age groups, the middle age-group (30-40 years) had a rate of convictions similar to the mean of the total cohort. In comparison, patients under 30 had a slightly higher level, and those over 40 had a slightly lower level. When data were stratified by pre-treatment levels of criminal convictions, the high conviction group had a substantially elevated risk of criminal convictions compared to patients with no or medium criminal convictions during the entire period of observation. The medium conviction group had a time continuous pattern similar to that of the overall mean, but with somewhat less crime out of treatment, i.e. a less obvious difference between in and out of treatment. Similar time-related patterns of convictions were found for all age subgroups.

## Discussion

The main finding in this study was that although conviction rates were broadly related to treatment status (in and out of treatment), changes in criminal behaviour tended to occur gradually and did not coincide in any precise way with entry to treatment or leaving treatment. Changes in criminal convictions were already evident *before* changes of treatment status. Reductions in criminal convictions were found in the period before entry (or re-entry) to OMT, and increases in criminal activity were found in the months prior to treatment interruption. As in other studies [9,13], the rates of criminal convictions continued to be relatively low when patients were in treatment, whereas outside of treatment the rates of criminal convictions continued to be relatively high. Although different levels in criminal convictions were found both before and after treatment entry for patients differing in age, gender and pre-treatment convictions, the same overall pattern of change was evident for all sub-groups.

Criminal convictions may be considered as a proxy on other types of problem behaviour, such as intensive substance abuse [30,31] and previous studies have reported that sizable numbers of treatment seeking people began making changes in substance use *prior* to treatment entry [32,33]. In a study investigating pre-treatment abstinence rates among treatment seekers, it was found that 45% of the patients reported stopping substance use prior to entering treatment [32]. Several factors may have contributed to the observed changes that occurred in criminal convictions prior to OMT. Individual factors such as problem recognition, awareness of need for change, willingness to receive treatment and commitment to participate in treatment may also have acted as factors affecting treatment engagement and reduction or cessation of drug use [34,35]. By actively applying for treatment, a process of change may have been initiated and supported, which may have led to less drug use and consequently less criminal convictions. Also, the changes may have been indirectly related to treatment: the reduction that was found in levels of criminal convictions in the months before initiation to treatment, corresponds approximately to the waiting list period, i.e. the period between application and treatment start. During the process of applying for OMT, patients may have established contact with the health and social services, and, for example, set up for long-term living arrangements [22]. More stable living and social conditions may have contributed to the reduction in criminal convictions prior to treatment initiation.

OMT is offered as a long-term or lifelong treatment. In practice, however, interruptions of treatment are common [16,36], and premature dropout is a usual problem facing patients, practitioners, and clinical researchers [17]. In our study, more than 35% of patients dropped out of treatment during the observation period, and some had multiple treatment episodes with entering and discontinuation of treatment. During the months prior to discontinuing treatment, the risk of criminal convictions gradually increased, and reached a higher level outside of treatment. The first few weeks after treatment cessations have been described as a *critical period*[37] both in terms of relapse to drug use and risk of overdose mortality [37-39]. The findings from the present study showed that the period of increased risk can be seen to precede the discontinuation of treatment. The elevated risk of convictions prior to treatment termination may be an indication of more chaotic personal circumstances at this time, resulting in increased drug use and as a consequence increased criminal activity.

Drug users may experience a number of incomplete or discontinued treatment attempts before being retained in long term maintenance treatment. Patients who have multiple episodes of maintenance treatment have been found to stay in treatment for progressively longer periods in later episodes [40], and readmission to more than one treatment episode may be protective of overdose mortality [39]. In the present study, patients with several treatment episodes showed significant reductions in criminal convictions prior to re-entering treatment as when entering treatment for the first time. This suggests that services should be prepared to readmit patients with previous incomplete or failed treatment attempts. Treatment policies that seek to restrict readmission to maintenance treatment may be counterproductive. Equally, clinical staff should be alert to the fact that there may be increased levels of criminal convictions before leaving treatment with attendant risks of increased drug use, morbidity, and drug related mortality in the period prior to treatment drop out.

The finding that changes in criminal convictions often occurred prior to changes in treatment status suggests that the factors associated with criminal activity may be, at least in part, independent of the treatment effects, and that such factors are located outside of the immediate context of treatment. This is consistent with other studies that have found reductions or cessation of substance use prior to treatment entry [32,33], and with studies that have shown the powerful effects that psychosocial factors can have upon relapse to illicit drug use during treatment [41].

### Limitations

Certain limitations of this study should be taken into account. Our study did not include data on self-reported criminal activity. Official crime records are known to underestimate the actual frequency of criminal activity, and crime statistics are considered a crude measure of heroin users’ involvement in crime. However, since this study investigates *changes* in criminal convictions during different phases of treatment, the patterns of criminal convictions are more important than the actual prevalence of criminal activity. Interpretation of our findings also needs to take account of potential confounding factors. Our study did not include data on whether patients spent time in prison, hospital or other institutions, or were in some other contact with other treatment services during periods of observation. Also, information was not available on whether patients ended treatment for non-compliance with programme requirements, or were voluntarily discharged. Important strengths of our study were having a complete national cohort of OMT patients, with no loss of patients during follow-up, and having a long observation period of nine years. Complete lists of patients allowed us to investigate the time periods when patients were outside of treatment. The criminal records have a further advantage in that they do not have gaps in the recording of criminal events throughout the lengthy study period. As all criminal offences were recorded for the specific date of the criminal offence, it was possible to calculate rates of criminal convictions on a day-to-day basis throughout the total observation period.

Despite the study limitations our results add to the understanding of the changes in behaviour that may occur among drug users seeking and receiving opioid maintenance treatment. The changes in problem behaviours that occur after entry to opioid maintenance treatment have been extensively studied. The changes that occur prior to changes in treatment status (entry to or leaving treatment) have received relatively little research attention.

The finding that changes in criminal convictions often occurred prior to changes in treatment status suggests that the factors associated with criminal activity may be independent of the treatment effects, and that such factors are located outside of the immediate context of treatment. This is consistent with other studies that have found reductions or cessation of substance use prior to treatment entry [32,33], and with studies that have shown the powerful effects that psychosocial factors can have upon relapse to illicit drug use during treatment [41].

### Conclusions

The results of the present study suggest that an important purpose of treatment may be to consolidate rather than to initiate behaviour change. The treatment of drug addiction is always a collaborative enterprise and drug problems cannot be treated without the co-operation and commitment of the patient [42]. More generally, the results support the utility of a broader temporal perspective upon the process of behaviour change that is not associated exclusively with the effects of the treatment intervention.

## Competing interests

The authors declare that they have no competing interests.

## Authors’ contributions

AB prepared the files for statistical analysis and drafted the paper. JR developed the model for calculating the time-continuous crime probability, preformed the statistical analysis regarding this model and took part in the discussion leading to the paper and writing up. SS prepared files for statistical analysis and participated in statistical analyses, discussion of the paper and writing up. MG participated in the study development, discussion of the paper and writing up. HW initiated the study and took part in the discussions leading to the paper and writing up. TC was project manager for the study, took part in the data collection and participated in statistical analysis and manuscript preparation. All authors have contributed to revising the manuscript and approved the final version to be published.

## Pre-publication history

The pre-publication history for this paper can be accessed here:

http://www.biomedcentral.com/1471-244X/13/262/prepub
